# Evolution of Gluten Content in Cereal-Based Gluten-Free Products: An Overview from 1998 to 2016

**DOI:** 10.3390/nu9010021

**Published:** 2017-01-03

**Authors:** María Ángeles Bustamante, María Pilar Fernández-Gil, Itziar Churruca, Jonatan Miranda, Arrate Lasa, Virginia Navarro, Edurne Simón

**Affiliations:** Gluten Analysis Laboratory of the University of the Basque Country, Department of Nutrition and Food Science, University of the Basque Country (UPV/EHU), Vitoria 01006, Spain; marian.bustamante@ehu.es (M.Á.B.); mariadelpilar.fernandez@ehu.es (M.P.F.-G.); itziar.txurruka@ehu.es (I.C.); jonatan.miranda@ehu.es (J.M.); arrate.lasa@ehu.es (A.L.); virginia.navarros@ehu.es (V.N.)

**Keywords:** gluten-free, cereal based foodstuff, gluten content evolution, ELISA, European regulation

## Abstract

The treatment of Celiac disease consists in a strict lifelong gluten-free (GF) diet. As the ingestion of small amounts can have damaging complications, there has been an ongoing discussion regarding the safe threshold for dietary residual gluten. The aim was to analyze the evolution of gluten content in cereal-based GF foodstuffs (*n* = 3141) from 1998 to 2016 measured by the enzyme-linked immunosorbent assay (ELISA) technique. Eight categories were defined: flours, breakfast cereals/bars, bakery, pasta, breads, dough, snacks, and yeasts, and these were divided into GF labeled-foods (GF-L) or reportedly GF foodstuffs, but not certified (GF-NC). Gluten-detection was decreased over time in line with the evolving European regulations about food information and gluten content claims. This decline started sooner in GF-L products than in GF-NC. As a whole, gluten was detected in 371 samples, with breakfast cereals/bars being the most contaminated group. Snacks and yeasts changed from being high gluten-detected samples to being totally GF over the years. The downside is that, of contaminated samples, those in the low levels of gluten detection range have decreased while flour samples containing over 100 mg/kg gluten have risen in the 2013–2016 period. Obtained data confirm that GF cereal-based foods are becoming safer but gluten control must be maintained.

## 1. Introduction

The only treatment for Celiac disease (CD) is the exclusion of gluten-containing cereals (e.g., wheat, rye, barley, and other closely-related cereal grains) and their derivatives in a strict lifelong gluten-free (GF) diet, achieving complete remission of symptoms. However, the ingestion of small amounts of gluten (which is called dietary transgression) can have serious and damaging complications [1]. For the majority of the individuals affected, intakes below 10 mg/day are unlikely to cause histological changes, while some authors have found that daily exposure to 50 mg/day is likely to damage intestinal mucosa [2,3].

Maintenance of a reliable gluten-free diet is a challenge, due to the fact that gluten is present in many more forms than just flours, bread, pasta or other cereal derivatives. Firstly, inherently gluten-free grains, such as rice, maize, quinoa, buckwheat, millet, or sorghum can be contaminated with gluten at different steps during their cultivation and processing, such as, crop rotation, milling, transportation, or handling. Furthermore, hidden sources of this protein can be commonly consumed because gluten is also widely used in several types of foodstuff as a thickener, flavour enhancer, emulsifier, filler, and fortification ingredient [4]. 

Taking the above into account, it has been difficult to establish a secure cutoff for residual gluten amount in GF products. In fact, for many years the standards of the Codex Alimentary Commission (the international organization founded by Food and Agriculture Organization and the World Health Organization) for gluten-free products dated back to 1979. At that time, Codex stated in the GF labelling purposes that products could be labelled gluten-free when total nitrogen content from the protein gluten did not exceed 0.05 g per 100 g of dry food, which was established as 200 mg/kg or ppm [5].

In 2008 The Codex standard for “foods for special dietary use for persons intolerant to gluten” [6] and the European Commission (Commission Regulation (EC) No. 41/2009) [7] introduced compositional and labelling standards that set levels of gluten for foods claiming to be either “gluten-free” (less than 20 mg per 1 kg food or 20 ppm) or “very low gluten” (less than 100 mg per 1 kg food, also expressed as 100 ppm). A similar rule for gluten-free labeling was established by the Food and Drug Administration (FDA) in 2013 [8]. 

Nowadays, Regulation (EC) No. 41/2009 has been repealed and these levels are supported by the Commission Implementing Regulation (EU) No. 828/2014. This regulation explains that different gluten sensitivity levels vary among people with gluten intolerance over a restricted range and that, on this basis, a food market with different low levels of gluten, always within that range, should be possible. Thus, this new standard allows the inclusion of food information for consumers accompanied by the statements “suitable for people intolerant to gluten” or “suitable for celiac” either for “gluten-free” or for “very low gluten” foods [9]. Nevertheless, considering that individual gluten sensitivity of celiac people is not commonly known, the general recommendation is to consume foodstuffs with the lowest gluten content and, thus, those advisory statements could be misleading among celiac consumers. In fact, the Association of European Celiac Societies (AOECS), only licences the use of the Cross-Grain symbol—quality mark—to manufactured products containing less than 20 mg/kg, that is, “gluten-free”. 

Although the market demand for GF food products is long-established, in recent years growing consumer need for GF foods has led to an increased development of these products. Therefore, the food industry has responded by improving its offer with new formulas of cereal-based GF foods [10]. To this end, as the removal of gluten from gluten-containing grains presents considerable technical difficulties and economic constraints, some celiac organizations have encouraged manufacturers of gluten-free-rendered foods towards the use of trademarks, such as the above-mentioned Crossed Grain symbol. Producers interested in using these trademarks should follow technical requirements for licensing. These include good manufacturing practices and Hazard Analysis of Critical Control Points (HACCP), thus ensuring the avoidance of gluten contamination during all stages of production, storage, transportation, and handling. Nevertheless, there are food manufacturers that decide not to include any quality mark in the labels of gluten-free products, although these, in fact, appear to be free of gluten based on a review of the list of ingredients contained.

In order to ensure consumer safety, it is necessary to evaluate gluten content in foods for special dietary use as gluten-free foodstuffs. Moreover, regulations like (EU) No. 1169/2011 require the declaration of cereals containing gluten even in unpackaged foodstuffs [11]. The few studies carried out in Europe and the USA have revealed a variety of gluten contamination (from 0.5% to 37% of the samples analyzed had over 20 mg/kg of gluten) [1,12]. Those studies were carried out over particular time-periods, but currently there is no information about the evolution on this prolamin content among the most commonly consumed cereal-based GF products over the years. The objective of this study was to analyze the changes in gluten content of these GF foodstuffs from 1998 to 2016. This overview would provide information for practitioners or CD patients about the reliability of gluten-free labeled products, as well as the potential safety of GF rendered products over the years.

## 2. Experimental Section

### 2.1. Food Samples

A number of samples (3141) of cereal-based GF foods sold in Spain were selected for gluten analysis from 1998 to 2016. Sampling was performed according to the production of cereal based GF products by food companies linked to guarantee marks, or by food safety control programs organized by health authorities or celiac associations. In the case of some samples, the same food products made by the same companies were analyzed in different years. 

These products were divided into two subgroups: either GF-labelled foods using a quality mark (GF-L) or foods assumed to be this on the basis of gluten-free ingredient list. This second group can be considered as reportedly GF, but not certified, as such (GF-NC).

Depending on the food characteristics, the samples were further sorted by eight categories flours, breakfast cereals/bars, pasta, breads, dough/pastry/pizza, bakery, snacks and yeasts. Although yeasts are not a final product, these samples were included as a regular raw material in bakery foods (Table 1).

### 2.2. Gluten Analysis by ELISA Techniques

Gluten content was studied by enzyme-linked immunosorbent assay (ELISA), as it is the currently accepted technique for gluten detection in foodstuffs [6]. During the period 1998–2016, two different methods have been used. The main differences between them are based on (1) the employment of different specificity antibodies; (2) diverse extraction methods; and (3) different reference materials or standards used for the assay calibration. Both methods have been recommended by organizations, such as Codex Alimentarius and/or AOAC International [13]. 

From 1998 to 2001, gluten was extracted using 40% aqueous ethanol solution. In this period gluten detection was performed using the commercial ELISA test Transia Plate Gluten (Diffchamb, Lyon, France), approved by AOAC International (method 991.19). This test is based on the anti ω-gliadin antibody (also called, 401.21) [13,14]. The reference material included in the kit was lyophilized gliadin extracted from bread wheat flour.

From 2001 to 2016, analysis were carried out using a RIDASCREEN^®^ Gliadin kit, (R7001, R-Biopharm AG, Darmstadt, Germany), approved by AOAC International (method 2012.01), INGEZIM gluten (R.30.GLU.K.2, Ingenasa, Madrid, Spain) and INGEZIM gluten Quick kit (R.30.GL2.K.2, Ingenasa, Madrid, Spain). All of these are based on the monoclonal secalin antibody R5. This detects gliadin fractions of wheat and corresponding prolamins from rye (secalins) and barley (hordeins), whereas prolamins from oats, maize, and rice are not detected [15]. Using these commercial kits, extraction was, in general, carried out using a 60% aqueous ethanol buffer containing reducing agents such as 2-mercaptoethanol. For those samples containing tannins and polyphenols like chocolate, cocoa, millet, etc., a special extraction procedure, consisting of adding the sample and different proteins in the same quantity, was carried out. When using the Ridascreen kit, skim milk powder (food quality, Nestlé España S.A., Barcelona, Spain) was added to the sample, whereas with Ingezim ones, gelatin from fish skin (SIGMA, St. Louis, MO, USA) plus polyvinylpyrrolidone (PVP) (SIGMA, St. Louis, MO, USA) were used (16.7% PVP in the sum sample and gelatin). In all cases the extraction was then completed following the general extraction procedure.

Prolamin Working Group (PWG) gliadin solutions are included as standards for preparing the calibration curve. This gliadin has been prepared from 40 different European wheat varieties by the European Working Group on Prolamin Analysis and Toxicity [16].

No significant differences were found among the different methods used in terms of false negatives or positives or detected (gluten) content range [17]. During the period from 1998 to 2008 the analyses were carried out according to the kit’s manufacturer instructions. The Transia Plate Gluten kit fixed the limit of quantification (LQ) at 10 mg/kg, whereas R5 antibody-based kits fixed the LQ at 5 mg/kg. Furthermore, internal validations were made to assure these assays. Since 2009 all analysis have been carried out using the ENAC (Spanish National Accreditation Body) accredited method 774/LE1626 according to ISO 17025 International Standards (ISO, 2005), which is based on the R5 antibody. The quantitative method was validated in terms of precision (repeatability and reproducibility), accuracy, and LQ. Repeatability (intra-day) showed a relative standard deviation (RSD) of 15%, while reproducibility (inter-day) obtained a RSD of 20%, and accuracy, calculated as recovery, was in the range 67%–115%. The limit of quantification (LQ) was determined at 5 mg/kg.

### 2.3. Statistical Analysis 

Statistical analyses of our results were performed by using the IBM SPSS statistical program 21 (IBM Inc., Armonk, NY, USA). The *χ*^2^ test followed by multiple comparisons (Bonferroni correction) was performed to determine differences in frequencies of categorized variables between groups. *p*-values < 0.05 were accepted as statistically significant. 

## 3. Results

In total, 3141 GF products, analyzed from 1998 to 2016, were divided into eight categories. Among these categories, bakery, flours, and bread were the most frequently analyzed samples (with 905, 564, and 498 sample numbers, respectively). As a whole, gluten was detected in 371 samples (Table A1). Yeasts and breakfast cereals/bars food groups represented the highest proportion of gluten-detected samples with 22.2% (8/36) and 21.5% (73/339), respectively. 

**Figure 1:**
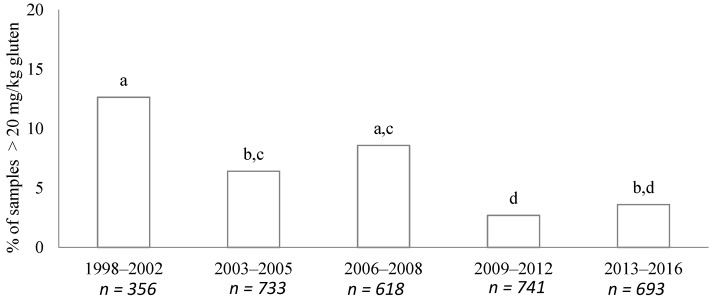
Evolution of gluten-containing samples (>20 mg/kg), sorted by five periods (1998–2002, 2003–2005, 2006–2008, 2009–2012, and 2013–2016). Data are expressed as the percentage of total samples analyzed in each period. Bars not sharing a common letter (a, b, c, d) are significantly different (*p* < 0.05).

A decrease in gluten-detected samples (>20 mg/kg of gluten) was observed over the years (Figure 1). The evolution of gluten-detected products from 1998 revealed that there were three different periods in relation to gluten content. The first period was from 1998 to 2002, the second period from 2003 to 2008, and the last one from 2009 to 2016. 

In the 1998–2002 period, the GF market was small and many of the foodstuffs contained gluten traces. In that period, 356 samples were analyzed and a 30% (107/356) contained detectable gluten. By food groups, percentages of contamination samples (>20 mg/kg of gluten) were as follows: breads, 17.5% (7/40; flours, 16.7% (8/48); bakery, 13.2% (16/121); breakfast cereal/bars, 11.3% (8/71); pastry/dough, 10.0% (2/20); pasta, 7.8% (4/51); snacks, 0% (0/4); and yeast, 0% (0/1). Six of forty bread samples analyzed contained more than 100 mg/kg of gluten.

Table 2 shows the evolution of gluten-detected samples from 2003 to 2016, according to three different gluten quantity intervals proposed by Regulation No. 828/2014 (gluten-free ≤20 mg/kg, very low gluten 21–100 mg/kg, and out of labelling >100 mg/kg). In the case of flour group, a progressive diminution in the percentage of gluten-free and very low gluten samples was observed among the detected-gluten samples. Meanwhile the ratio of samples not suitable for celiac people was continuously increasing from 2003 to 2016. Taken as a whole, the same tendency was revealed in all analyzed food groups (Table 2). The percentage of samples whose gluten content was in the range of 5–20 mg/kg from all gluten detected samples was 55% (57/104) in 2003–2005 time period and reduced to 19% (6/31) in 2013–2016 period. By contrast, in the case of samples over 100 mg/kg of gluten the percentages increased from 13% (14/104) to 58% (18/31) for the same time periods.

**Figure 2:**
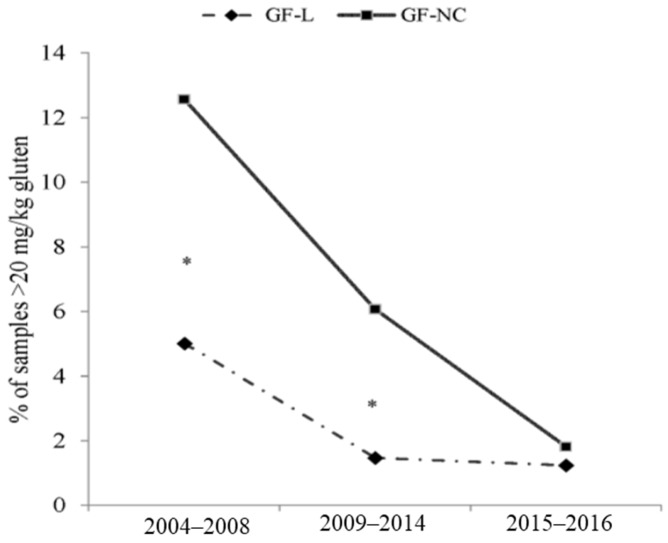
Evolution of gluten-containing samples (>20 mg/kg) sorted by three time periods related to gluten regulation (2004–2008, 2009–2014, and 2015–2016). From the total number of 2614 samples, 1652 were gluten-free-labeled products (GF-L) and 962 reportedly gluten free, but not certified, products (GF-NC). Data are expressed as percentages of the total sample analyzed in each period. Significantly different time period are expressed as * (*p* < 0.05).

Until 2008 the snack and yeast food groups differed from the rest of the groups analyzed, with a higher percentage of samples over 100 mg/kg of gluten (Table 3). From 2008, these two groups dramatically reduced the percentage of samples not suitable for any statement on the product label. Therefore, after 2008, there was no difference between snack and yeast and the rest of the groups. In the 2009–2016 time period, only the flour food group showed a slightly greater percentage of samples not suitable for any label mark (5% (14/276) for flours vs. 1% (2/374) for bakery products) (Table 3). In order to relate the decrease of gluten-positive products in line with the changing of European regulations requiring information to be given about gluten content in foods, a specific analysis of gluten-free-labeled products (GF-L) and reportedly gluten free, but not certified (GF-NC), products was made (Figure 2). Considering that most of the samples analyzed were carried out from 2004 to 2016, this period was selected for the evaluation. The number of products studies for GF-L was 1652, and for GF-NC was 962. As it is indicated in Figure 2, at the start point there was a higher percentage of gluten-positive samples in GF-NC than in GF labeled ones (12.6%, 45/358 vs. 4.9%, 41/817). The comparison revealed differences between GF-L and GF-NC in 2004–2008 and 2008–2014 periods of time, but not in the last two years (Figure 2). 

## 4. Discussion

According to the Mintel’s report the GF product market not only represents one of the most prosperous markets in the field of food and beverages nowadays, but also offers positive perspectives in the near future, with a forecast growth of around 10% [10]. Apart from celiac people, other consumers as a result of cultural- or health-beliefs and dietary habits, are responsible for the growth of the GF food market [18]. 

For people with CD, the involuntary intake of gluten, apart from dietary transgressions of GFD, is probably one of the major reasons for symptom persistence. This unintentional intake could be for two main reasons: contamination of the foodstuff at some step of the manufacturing process, or inadvertent gluten intake due to misleading nutritional labelling. In order to protect the celiac population’s rights several laws have been put into place in the last decade. First, in 1979 the Codex adopted a standard for foods for special dietary use for people intolerant to gluten, which, later, in 2008, was revised and corrected [6]. This document set the definition of gluten-free foods as those containing less than 20 mg/kg. As mentioned, the terms gluten free (≤20 mg/kg) and very low gluten (21–100 mg/kg) are nowadays covered by 2014 legislation relating to GF foods [9]. Consequently, samples with gluten content over 100 mg/kg are not suitable for any statement on the product label.

Bearing this in mind, our results for products sold in Spain confirmed that rules implemented to control gluten content were effective. There was a marked cutoff year, 2008, with a strong reduction of the gluten-positive samples (>20 mg/kg of gluten). 

Other studies in Europe have been conducted in order to evaluate the gluten content of GF products. Before the cut-off year, Valdes et al. carried out research where a large miscellaneous group (*n* = 4454 samples) comprising gluten-free foods was analyzed [12]. They found that close to half of the samples contained detectable gluten whereas in that period (1998–2002), we found that nearly one third of the analyzed samples were contaminated (Figure 1). This discrepancy might be justified, at least in part, by the quantification limit (LQ) established because these authors set a lower LQ (3.2 mg/kg) than ours. Furthermore, they found a higher ratio of samples over 20 mg/kg than we did during the period of 1998–2002. It has to be taken into account that they analyzed many flours (rice, maize, oats origin) while we measured processed products. Indeed, in less processed categories such as bread or flours, we found a larger percentage of contaminated samples over 20 mg/kg, data that are closer to those obtained by Valdes et al. [12]. 

1998–2002 was a confusing time-period, due to the scarce European regulation in terms of gluten control. This allowed a high proportion of gluten contaminated samples in a low diversity GF product market. By contrast, from 2003 onwards, analyses performed by Gibert et al. for Italian, Spanish, German, and Norwegian samples indicated that, out of 205 samples, only one (0.5%) was over the gluten threshold [1]. These data are in the same line as our results obtained in the 2009–2012 period (2.7%), confirming that Codex revision implementation was efficiently followed by Central and Western European countries.

Nevertheless, celiac people cannot completely presume the foodstuffs on offer to be safe. The evolution of analyzed products revealed that, in general, when gluten is detected in samples nowadays, it is detected in higher quantities (<100 mg/kg of gluten) than 10 years ago. Considering that most of the analyzed products have to represent the basis of the diet, due to the fact that they provide carbohydrates and, thus, the main energy source, those gluten-contained products represent a real concern for celiac people. 

Additionally, it must be emphasized that before Codex revision (prior to 2008) snacks and yeast could be considered the most risky food for celiac people. Both showed elevated percentages of samples not suitable for people with celiac disease compared to the rest of analyzed food groups (12% for snacks and 21% for yeasts). However, after the revision, no more differences in samples containing more than 100 mg/kg among food groups were observed.

In the case of snacks, one possible explanation for this fact could be related to consumer preferences. A Nielsen global snacking report indicated that, nowadays, the GF aspect of a snack is very important for one-fifth of global respondents [19]. Traditionally maize has been the prime flour source to produce extruded snacks [20], despite the addition of wheat in some formulations. Taking into account the tendencies in snack preferences in recent years, they cannot be discounted as responsible for the gluten-contamination control improvement from 2008. Apparently, something similar could take place for bakery yeasts. Sugars are the source of yeast fermentation and, therefore, of bakery yeast production. Whether the origin of sugars is related to gluten containing cereals (rye, wheat, and barley) or not is a simple decision of yeast manufacturers. It might be said that the 2008 Codex revision made bakery yeast manufacturers aware of the need to reinforce their gluten control, probably changing the sugar source.

In terms of GF cereal products, as mentioned before, two cases apply to a gluten-free claim. On one hand, there are gluten-free foodstuffs produced by manufacturers which include a quality mark, label, or certificate of prolamin content below 20 mg/kg. Alternatively, there are unlabeled products that appeared gluten-free based on scrutiny of their listed ingredients [21]. Our results reveal that there were differences in both, GF labelled (GF-L), and reportedly GF, but not certified products (GF-NC), over time. Specifically during the 2004–2014 time-period, a higher rate of gluten-positive samples was detected in GF-NC samples than in GF labelled ones. However, after 2015 both groups of samples showed a similar range of positives.

After the revision of the Codex standard (2008), and basing on it, the European commission regulated the provision of food information to consumers with No. 1169/2011 [11]. Although this regulation was made in 2011, the deadline for its mandatory complementation was 13 of December 2014. It could be postulated that this brought about the closing of the gap between GF-labeled and GF-NC products in terms of positive samples.

It is worth noting the evolution of not certified GF products from 2004, during which time a constant and noticeable reduction in gluten-positive samples can be observed. Very much in line with this tendency, the literature reflects how research conducted in Europe and published in 2010, 2011, and 2013, detected decreasing ratios of gluten-positives samples (10.5%, 9.7%, and 0.5%, respectively) [1,22,23]. 

Outside Europe, other countries adopted similar rules in terms of gluten. With compliance date of August 2014, the USA Food and Drug Administration regulated the term GF as did European regulation [8]. Prior to 2014, in the USA, there was reported a high gluten detection in gluten-free grains, seeds, and flours but not in the labelled products (32% vs. 3.6%–5.1%) [21,24,25]. As in our study, there was a clear difference between both kinds of GF products before GF regulation. However, a study published in 2016 revealed that positive samples ratio for GF-NC products went down (4.9% from a total of 101) [26]. In view of the above, it seems that GF rule implementation in the USA was effective as the number of positive samples decreased not only among GF labeled products, but also in GF-NC ones.

As far as we know this is the first study that analyzes the evolution of gluten detection in GF products over a long period of time. Furthermore, data of gluten presence sorted by five ranges provide useful information for food safety authorities, manufactures, practitioners, and other related professionals working on gluten and CD from other countries, who do not set the gluten threshold at 20 mg/kg (Table A1). For instance, Australia and New Zealand, recently, set narrower regulations establishing that “gluten-free” foods must not contain detectable gluten [27]. However, it is necessary to consider that the non-standardized sampling of this research is not representative of the entire Spanish gluten-free cereal retail market. Due to this fact, information about the raw material in origin (rice, corn, quinoa, or others) of all of the samples was not collected. On the other hand, the categorization proposed in this research could not fit with other authors, limiting, at least in part, specific comparison. For instance, some authors include bread in bakery foods [28] and others defined other group, such as a convenience food category [29].

## 5. Conclusions

In summary, a tendency toward a reduction in the presence of gluten contamination in gluten-free rendered foods over the years has been observed. Our results confirm the effectiveness of European regulation in terms of gluten control for GF foodstuffs. Indeed, the significant drops which have taken place can be linked to European regulations about gluten content in food and, probably, to the involvement of the food industry over the years. In this context, the data obtained in recent years are reassuring and make grain-based foods more reliable products for the celiac population, but strict gluten control should be maintained.

## Figures and Tables

**Table 1:** Food categories of samples used for gluten quantification.

Category	Selected Examples
Flours	starches, baking mixes, all-purpose flours, grains and seeds
Breakfast cereals/bars	corn and other GF cereal pancakes, granola bars, soy/quinoa/almond/rice beverages, corn flakes, rice crisps, rice and quinoa waffle, muesli
Pasta products	macaroni, rices/multigrain/corn pasta, rice, lasagna sheets, semolina, noodles, tagliatelle, pasta with egg, with vegetables, fettuccini, cooked and dry pasta, organic pasta
Breads	baguettes, loaf, sliced or toasted bread, breadcrumbs, breadsticks, white/multi-grains/artisan/rustic bread, pita bread, crackers, wraps, bread rolls, ciabatta, bagels, hamburger buns
Dough/pastry/pizza	all types of pizza, pastry, croquettes, baked dough, wafers, pizza bases, all kind of sandwiches, cooked lasagna
Bakery	all types of cakes, chocolate/fruit/filled cookies, biscuits, muffins, cupcakes, scones, pies, donuts, sweet rolls, croissants, shortbread, sponge cake
Cereal-based snacks	salted/sweet popcorn, tortilla chips, pretzel cereal treats, cheddar/chili corn sticks, rice/corn triangles, fried corn nuts, baked corn snack with flavours (butter, ham, cheese, ketchup), flavour fried potato crisps, flavour rice and corn snack, crunchy/crispy/flavour crackers and bugles
Yeasts	bakery yeast and chemical leavening agents

**Table 2:** Time-period comparison of gluten-detected samples.

Food Group	Gluten Content (mg/kg)	Time Period			
		2003–2005	2006–2008	2009–2012	2013–2016
Flour	5–20	67 (6/9) ^a^	37 (11/30) ^a,b^	27 (3/11) ^a,b^	0 (0/10) ^b^
	21–100	22 (2/9)	40 (12/30)	18 (2/11)	20 (2/10)
	>100	11 (1/9) ^b^	23 (7/30) ^b^	55 (6/11) ^a,b^	80 (8/10) ^a^
Breakfast cereals/bars	5–20	72 (21/29)	40 (6/15)	33 (1/3)	33 (1/3)
	21–100	24 (7/29)	27 (4/15)	33 (1/3)	33 (1/3)
	>100	4 (1/29)	33 (5/15)	33 (1/3)	33 (1/3)
Bakery	5–20	57 (17/30)	38 (3/8)	67 (6/9)	67 (2/3)
	21–100	37 (11/30)	50 (4/8)	11 (1/9)	33 (1/3)
	>100	7 (2/30)	12 (1/8)	22 (2/9)	0 (0/3)
Pastry/dough	5–20	50 (3/6)	0 (0/3)	83 (5/6)	0 (0/1)
	21–100	33 (2/6)	33 (1/3)	17 (1/6)	0 (0/1)
	>100	17 (1/6)	67 (2/3)	0 (0/6)	100 (1/1)
Breads	5–20	100 (4/4)	60 (3/5)	0 (0/1)	27 (3/11)
	21–100	0 (0/4)	20 (1/5)	0 (0/1)	9 (1/11)
	>100	0 (0/4)	20 (1/5)	100 (1/1)	64 (7/11)
Pasta	5–20	24 (4/17) ^b^	78 (7/9) ^a^	33 (1/3) ^a,b^	0 (0/3) ^a,b^
	21–100	59 (10/17)	11 (1/9)	0 (0/3)	67 (2/3)
	>100	18 (3/17)	11 (1/9)	67 (2/3)	33 (1/3)
Snacks	5–20	40 (2/5)	40 (8/20)	67 (2/3)	-
	21–100	20 (1/5)	20 (4/20)	33 (1/3)	-
	>100	40 (2/5)	40 (8/20)	0 (0/3)	-
Yeasts	5–20	0 (0/4)	67 (2/3)	-	-
	>100	100 (4/4)	33 (1/3)	-	-
Total	5–20	55 (57/104) ^a^	43 (40/93) ^a,b^	50 (18/36) ^a,b^	19 (6/31) ^b^
	21–100	32 (33/104)	29 (27/93)	17 (6/36)	23 (7/31)
	>100	13 (14/104) ^c^	28 (26/93) ^b,c^	33 (12/36) ^a,b^	58 (18/31) ^a^

Notes: Data are expressed as percentage of gluten-content interval (5–20, 21–100, and >100 mg/kg) from total gluten-detected samples, analyzed by each period and categorized by food group. The numerical fraction of samples detected for each range, in each food group and in each time period are expressed in brackets. Percentages not sharing a common letter (^a, b, c^) are significantly different (*p* < 0.05).

**Table 3:** Comparison by food groups of samples under or over 100 mg of gluten per kg of product before and after 2008 year.

Time Period	Gluten Content (mg/kg)	Food Group							
		Flour	Breakfast Cereals/Bars	Bakery	Pastry/Dough	Breads	Pasta	Snacks	Yeasts
2003–2008	≤100	97 ^a,b^ (232/240)	97 ^a,b^ (171/177)	99 ^a^ (407/410)	97 ^a,b^ (96/99)	99 ^a^ (167/168)	97 ^a,b^ (145/149)	88 ^b,c^ (74/84)	79 ^c^ (19/24)
	>100	3 ^a,b^ (8/240)	3 ^a,b^ (6/177)	1 ^a^ (3/410)	3 ^a,b^ (3/99)	1 ^a^ (1/168)	3 ^a,b^ (4/149)	12 ^b,c^ (10/84)	21 ^c^ (5/24)
2009–2016	≤100	95 ^b^ (262/276)	98 ^a,b^ (89/91)	99 ^a^ (372/374)	99 ^a,b^ (172/173)	97 ^a,b^ (282/290)	97 ^a,b^ (110/113)	100 ^a,b^ (106/106)	100 ^a,b^ (11/11)
	>100	5 ^b^ (14/276)	2 ^a^ (2/91)	1 ^a^ (2/374)	1 ^a,b^ (1/173)	3 ^a,b^ (8/290)	3 ^a,b^ (3/113)	0 ^a,b^ (0/106)	0 ^a,b^ (0/11)

Notes: Data are expressed as percentage of gluten content interval (≤100 and >100 mg/kg) from total analyzed samples, categorized by each food group. The numerical fraction of samples detected for each range, in each food group and in each time period are expressed in brackets. Percentages not sharing a common letter (^a, b, c^) are significantly different (*p* < 0.05).

## Appendix A

**Table A1:** Summary of results obtained in gluten detection analysis from 1998 to 2016.

Food Group	Analyzed Sample Number	Gluten-Detected Samples	Gluten (mg/kg)				
			5–10	11–20	21–100	101–200	>200
Flours	564	75	10	17	21	11	16
Breakfast cereals/bars	339	73	22	22	15	6	8
Bakery	905	87	21	28	24	6	8
Pastry/dough	292	23	7	6	6	0	4
Bread	498	31	5	8	3	4	11
Pasta	313	45	8	13	14	4	6
Cereal based-Snacks	194	29	5	8	6	2	10
Yeasts	36	8	2	1	0	1	4
Total	3141	371	80	103	87	34	67

Notes: Data related to gluten quantification are expressed as number of samples.
